# Telehealth-related education and training: a systematic review of recent developments since the COVID-19 pandemic

**DOI:** 10.1080/10872981.2026.2692839

**Published:** 2026-10-15

**Authors:** SDC Catapan, Monica Taylor, Hans Hyun Seok Cheong, Jaimon T. Kelly, Sisira Edirippulige

**Affiliations:** a Centre for Online Health, The University of Queensland, Brisbane, Australia; b Centre for Health Services Research, The University of Queensland, Brisbane, Australia; c Medical School, Faculty of Medicine, The University of Queensland, Brisbane, Australia

**Keywords:** Telehealth, telemedicine, education, training, curriculum

## Abstract

The pandemic showed the potential of existing telehealth to increase healthcare access but also the need for healthcare workers to be competent and skilled in delivering care through this modality. We undertook a systematic literature review to identify telehealth-related courses developed since the start of the COVID-19 pandemic and summarised study characteristics, learning objectives, and course content. Articles describing telehealth-related training and education programs implemented between 2020 and 2024 were included. Papers were required to list specific program content or learning objectives for any educational level or type of health professional. Two authors independently extracted data. Quality of articles was assessed against the SQUIRE 2.0 guidelines. Quantitative data was summarised and reported using frequencies and percentages, learning objectives were mapped across the six Association of American Medical Colleges (AAMC) telehealth competency domains, and qualitative categories were thematically synthesised. Of 7547 articles identified, 51 were included. Most courses were implemented in the USA (82%), mostly targeted to undergraduate students (51%), and the number of articles per year increased over time. Most courses were developed by universities (53%), sometimes in collaboration with health services, and content was primarily applicable across multiple specialties. Participants nearly always evaluated the courses (90%). Content included basic knowledge and skills in telehealth, patient safety, and ethics, with learning objectives often on technology and communication. Only four programs included all AAMC telehealth competencies. This review highlights that telehealth education and training has expanded since the onset of the COVID-19 pandemic, however, current curricula are lacking experiential learning. Courses generally covered telehealth communication competencies in detail, but more content on the ethical and legal requirements, billing, physical examination, patient education, and eligibility for telehealth is required. For efficient resource use, standardised training can be applied across disciplines with addition of site or speciality-specific modules.

## Introduction

Telehealth involves the use of information and communication technologies in healthcare, including consultations between patients and healthcare workers [1]. Telehealth modalities include telephone and video consultations, also called virtual care, while digital health is an umbrella term that encompasses telehealth as well as other applications such as electronic records, artificial intelligence (AI), and clinical decision systems [2]. Telehealth has been around for the main part of modern medicine, with first instances of its use in Australia dating back to 2000, utilising a Telepaediatric model to encourage referrals from regional clinicians [3]. Two decades later, telehealth has had a major uptake worldwide due to the COVID-19 pandemic. The Australian public health system saw a 63-fold increase in telehealth services during the first year of the pandemic [4]. A similar trend was observed globally with nearly 11-fold increase in telehealth services in Scotland within the first two weeks of the pandemic [5], and 8.5-fold increase in the USA in the first three months [6].

The pandemic has shown the potential of telehealth to increase healthcare access but also the need for healthcare workers to be competent and skilled in delivering care virtually [7–9]. Training the workforce to deliver high-quality telehealth services remains one of the main challenges to scale-up [10,11]. Beyond clinical knowledge, healthcare professionals need to develop technological, communication, and interpersonal skills as well as awareness of telehealth benefits and limitations [12]. In response to the large expansion of telehealth in the USA, the Association of American Medical Colleges (AAMC) defined six core telehealth competency domains for medical residents and professionals which are: patient safety and appropriate use; data collection and assessment; communication; ethical practices and legal requirements; technology; and access and equity [13].

Similarly, the Australian Medical Council, in collaboration with the Australian Digital Health Agency, has created a digital capability framework to assist providers in building a digitally skilled medical workforce. The framework covers key domains such as health system innovation, professionalism and inter-agency collaboration, integrated health settings and access, as well as appraisal and risk of digital health tools and technologies [14].

Despite an increasing number of articles describing the specific telehealth skills necessary to different healthcare occupations [8,15], there is currently limited information about the type of education and training available to clinicians and future health workers. Those that have been described are mainly mapped and summarised before the pandemic [16,17]. Therefore, this study aims to evaluate the current literature on telehealth-related courses developed after the start of the COVID-19 pandemic and summarise characteristics, learning objectives, content, and delivery methods.

## Methods

### Study design

This systematic review of literature followed the Preferred Reporting Items for Systematic Reviews and Meta-Analyses (PRISMA) guidelines [18]. The protocol for this review was registered with the Open Science Framework (https://osf.io/ywntr).

### Search strategies

To clearly outline the search strategy, the Population Concept Context (PCC) framework was used to refine the targets of the search. Population (P) included all clinicians working or training in healthcare, regardless of telehealth experience. The concept (C) was video and telephone consultations, often called telehealth services, used by clinicians to facilitate interactions with patients. And the context (C) included telehealth-related academic education or training offered at undergraduate or postgraduate level, training, professional learning, continuous education or vocational continuing professional development (CPD).

The search strategy was developed by combining search terms identified from preliminary searches and related to the definitions used in the PCC framework (full search strategy available in Appendix A). Searches were conducted in September 2024. Databases searched were MEDLINE via PubMed, Embase, CENTRAL via Cochrane, CINAHL, PsycINFO, Scopus, and Web of Science.

### Eligibility criteria

Articles that focused specifically on the delivery of telehealth-related training and education at an academic level (undergraduate or postgraduate students), professional learning, continuous education or vocational continuing professional development (CPD), which described the content or learning objectives were included. Clinicians were considered all healthcare professionals that provide clinical care in any setting, with or without experience or training in telehealth. Both healthcare clinicians and students included medical specialists, doctors, allied health and nurses.

Telehealth courses entailed all training or education programmes tailored to increase the skill or proficiency in using the technology involved in telehealth, conducted in more than one single session. Individual (one off) training sessions which were not part of a telehealth education program, including telehealth simulation sessions using Objective Structured Clinical Exam (OSCE), or telehealth training programmes that did not specify the delivered content or learning objectives, explicitly or not, were excluded.

Articles that described the use of telehealth for patient or health professional's education (telementoring), measuring health professionals' skill or competency to conduct telehealth, describing the implementation of a telehealth service with minimal training attached, or describing satisfaction or perception towards a telehealth education program, without providing any information about the education program itself, were all excluded. Grey literature, literature reviews, and commentaries were also excluded, but any eligible papers within those were considered for inclusion.

Due to recent changes in the telehealth field in response to the pandemic, we restricted the timeframe to post-COVID articles (data collected 2020 onwards), and articles had to be available in full text in English for researchers to analyse.

### Screening

Database searches were conducted by the third author (HC) with the support of a university librarian and the first author (SC). Results were exported to Covidence to remove duplicates and for screening. All papers were reviewed independently by two authors (HC, SC, MT) and conflicts were resolved by discussion until consensus.

### Data extraction

Characteristics of the studies were extracted and tabulated in Excel, including characteristics of the study (author, year, country, study design); participant (demographics, specialty, sample size); course (learning objectives, content, delivery method, developer, level of education); and evaluation (outcome measure, results and implementation barriers and facilitators).

### Quality assessment

Quality assessment was conducted by two reviewers independently according to the guidelines by the Revised Standards for Quality Improvement Reporting Excellence (SQUIRE 2.0) checklist [19].

### Data analysis

After finishing data extraction, each column of the data extraction spreadsheet was checked for consistency and either discussed or reviewed by another author, if needed. Aspects of identified courses that could be quantified, such as year and country of publication, study design, participant demographics and sample size, were summarised and reported using frequencies and percentages. Learning objectives explicitly mentioned in the studies were mapped across the six domains of the AAMC telehealth competency domains [13] and also reported using frequencies and percentages. For other qualitative categories, thematic synthesis was used to summarise findings [20].

## Results

### Screening

From 7547 unique results initially identified through the searches, a total of 51 articles met all criteria and were included in this review. The full article selection and screening process is shown in Figure 1.

**Figure 1.**
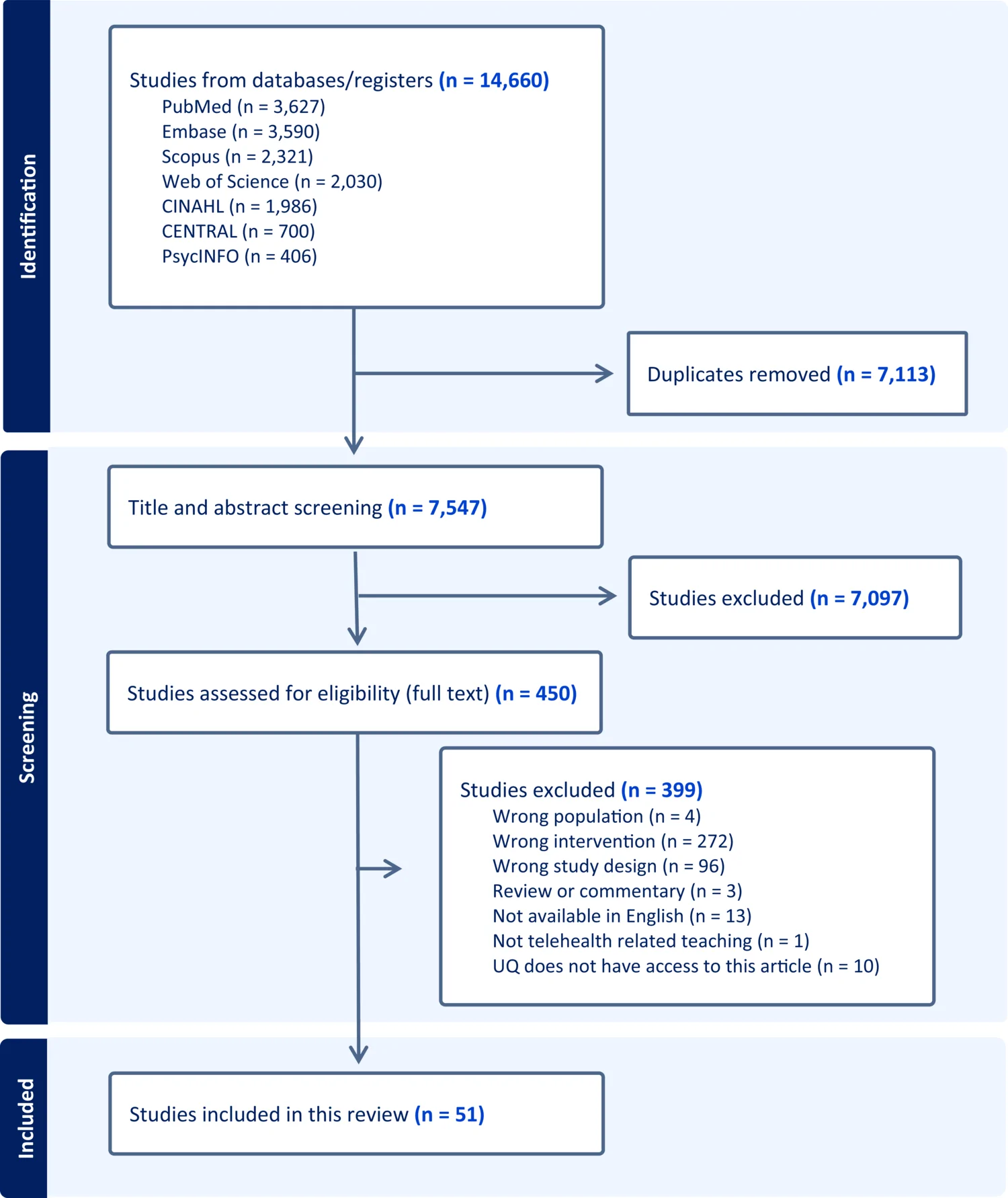
PRISMA flow diagram of study screening process, including: identification, screening, and inclusion phases with numerical counts. Finally, 51 studies were included in the review.

### Quality assessment

Of the 51 included articles, seven (14%) had a full score of 18 reporting all items from the SQUIRE 2.0 guidelines [21–27]. The average number of items reported was 15 out of 18. The description of quantitative and qualitative methods was frequently overlooked, with 29 articles (57%) omitting this and simply mentioning a survey was used with its findings. Only 59% of articles (*n* = 30) adequately interpreted their findings, with many not comparing their results with existing literature. Sources of funding for the work (*n* = 32, 63%) and ethical considerations (*n* = 33, 65%) were the next lowest reported items. The problem description was sufficiently reported by all (100%) of the articles, which was often the need for improved virtual care training in response to the COVID-19 pandemic and increased use of telehealth.

### Study characteristics

Most curricula were implemented and evaluated in the USA (*n* = 42 studies, 82%), with two articles from Canada (4%) and one study each from Australia, Brazil, Israel, Japan, Malaysia, Scotland, and the UK. The number of studies per year increased over time with five (10%) included from 2020, 12 (24%) from 2021, 13 in 2022 (25%), 14 (27%) in 2023 and 7 (14%) until September 2024. Most courses were offered to undergraduate students only (*n* = 26, 51%), whereas 22 (43%) were offered to postgraduate students, including doctors in residency and professional development, and 3 (6%) targeted both undergrad and postgraduate students. Table 1 summarises main study characteristics including targeted audience, specialty or discipline of focus and course delivery method.

**Table 1.** Study characteristics (*N* = 51).

First author, year	Country	Targeted audience	Level of education	Specialty/discipline	Course delivery method
Abraham et al. [28]	USA	Medical students	Undergraduate	Internal Medicine	Online synchronous (live) and asynchronous
Abrams et al. [29]	USA	Medical students (3rd year)	Undergraduate	Not reported	Online synchronous (live) and asynchronous
Adamkiewicz et al. [30]	USA	Medical students (preclinical)	Undergraduate	Chronic care (multidisciplinary)	Not reported
Anawati [31]	Canada	Medical students	Undergraduate	Medicine	Not reported
Arends et al. [32]	USA	Nurse students (final year)	Undergraduate	Family Nursing	Online synchronous (live) and asynchronous
Aziz et al. [33]	USA	Medical residents	Doctor in residency	Ophthalmology	Online synchronous (live) and asynchronous
Bajra et al. [25]	USA	Medical students (3rd and 4th year)	Undergraduate	Family Medicine	Online synchronous (live)
Bajra et al. [34]	USA	Medical students and family medicine residents	Undergraduate/Doctor in residency	Family Medicine	Online asynchronous
Baliko et al. [35]	USA	Nurse students	Undergraduate	Family Nursing	Online synchronous (live) and asynchronous
Berrier et al. [35]	USA	Nurse students (2nd year)	Undergraduate	Family Nursing	Online asynchronous and in-person
Booth et al. [27]	Scotland	Medical students (final year)	Undergraduate	Obstetrics and gynaecology	Online synchronous (live) and in person
Chike-Harris et al. [36]	USA	Registered nursing students	Postgraduate/Professional development	Psychiatric-mental health nurse practitioner	Online asynchronous and in-person
Costich et al. [37]	USA	Medical residents	Doctor in residency	Paediatrics/Primary care	Online synchronous (live)
Creese et al. [23]	USA	Pharmacology students (1st, 2nd and 3rd year)	Undergraduate	Pharmacology	Online synchronous (live) and asynchronous
Darnell et al. [38]	USA	Doctor of Pharmacology students (2nd and 3rd year)	Postgraduate	Pharmacology	Not reported
DeFoor et al. [39]	USA	Nurse students (final year)	Undergraduate	Women's health/Post-natal care	In-person
Farrell et al. [40]	USA	Medical students	Undergraduate	Not reported	Online asynchronous
Frankl et al. [41]	USA	Medical students	Undergraduate	Medicine (multidisciplinary)	Online asynchronous
Gafni-Lachter et al. [42]	Israel	Healthcare providers (Occupational therapists, physical therapists, speech therapists, social workers and psychologists)	Postgraduate	Paediatric (multidisciplinary)	Online
Guizado de Nathan et al. [43]	USA	Medical students (1st year)	Undergraduate	Medicine/Interpreter services	Online synchronous (live) and asynchronous
Gunner et al. [44]	UK	Medical students	Undergraduate	Not reported	Online synchronous (live) and asynchronous
Ha et al. [45]	USA	Medical residents	Doctor in residency	Family Medicine	Online synchronous (live)
Hatcher et al. [46]	USA	Communication Sciences and Disorders students	Undergraduate	Speech pathology	Online asynchronous and in-person
Hebert et al. [47]	USA	Nurse students (varying levels)	Undergraduate	Nursing	Not reported
Homeward-Pascal et al. [48]	Canada	Registered nursing students	Postgraduate/Professional development	Oncology	In-person
Jones et al. [49]	USA	Registered nursing students	Postgraduate	Nursing (multiple specialties, including midwifery)	Online synchronous (live) and asynchronous
Kazawa et al. [50].	Japan	Nurse students (final year)	Undergraduate	Chronic care	Online
Keyserling et al. [51]	USA	Medical residents	Doctor in residency	Internal Medicine/Primary Care	Not reported
Lesselroth et al. [52]	USA	Medical students (3rd year) and Physician assistant (>6 months) students	Undergraduate/Postgraduate	Community Medicine/Teledermatology	Online asynchronous and in-person
Lesselroth et al. [53]	USA	Medical students (3rd year) and Physician assistant (2nd year) students	Undergraduate	Medicine and physician assistant	In-person
Liew et al. [24]	Malaysia	Medical students (4th and 5th years) and Medical graduates (1st year)	Undergraduate/Doctor in residency	Medicine	Online synchronous (live) and asynchronous
Love et al. [54]	USA	Registered nursing students	Postgraduate	Nursing	Online synchronous (live) and asynchronous
Lowe et al. [55]	USA	Healthcare providers	Postgraduate/Professional development	Not reported	Online asynchronous
MacNaul et al. [26]	USA	Registered Behavioural Technicians	Postgraduate	Behaviour technician	Online synchronous (live)
McConnell et al. [56]	USA	Medical residents	Doctor in residency	Internal Medicine/Paediatrics	Online synchronous (live)
Moore et al. [57]	USA	Registered nursing students	Postgraduate/Professional development	Nursing (outpatient)	Online asynchronous and in-person
Mulcare et al. [58]	USA	Medical students	Undergraduate	Not reported	In-person
Ostrovsky et al. [22]	USA	Medical, nurse practitioner and physician assistant students	Undergraduate	Medicine, nursing and physician assistant	Online
Rutledge et al. [59]	USA	Healthcare students (various)	Undergraduate	Multidisciplinary	Online synchronous (live) and asynchronous
Safdieh et al. [60]	USA	Medical students	Undergraduate	Multidisciplinary	Online
Samuels et al. [61]	USA	Medical residents	Doctor in residency	Paediatrics	Online asynchronous
Savage et al. [62]	USA	Medical residents	Doctor in residency	Primary Care	Online
Schinasi et al. [63]	USA	Healthcare providers (physicians, psychologists, social workers, nutritionists, genetic counsellors, case managers, and others)	Professional development	Paediatrics	Not reported
Schroeder et al. [64]	USA	Healthcare providers	Postgraduate	Tailored towards mental health	Online synchronous (live)
Severini et al. [21]	Brazil	Medical residents	Doctor in residency	Paediatrics	Not reported
Sheikh [65]	USA	Registered nursing students	Postgraduate	Family Nursing	Online asynchronous and in-person
Shin et al. [66]	USA	Medical students (3rd year)	Undergraduate	Medicine/Interpreter services	Online asynchronous
Taylor et al. [67]	USA	Medical students	Undergraduate	Osteopathy	Online synchronous (live), asynchronous and in-person
Taylor et al. [68]	Australia	Rural doctors	Postgraduate	Rural medicine	Online synchronous (live)
Uthappa et al. [69]	USA	Healthcare students (various)	Undergraduate	Multidisciplinary	Online synchronous (live)
Watt et al. [70]	USA	Medical residents	Doctor in residency	Anaesthesiology	Online synchronous (live)

### Study design

As part of the inclusion criteria, all articles had some form of description of their curriculum (e.g., descriptive case study). Five articles only provided descriptive analysis [31,39,63–65], while all the remaining also evaluated their implemented curricula. Most studies (*n* = 26, 51%) used a pre and post-test study design, 14 (27%) used cross-sectional survey methods, and four (8%) only employed post-survey methods. There was also one article that evaluated the training using a randomised-controlled trial [61] and one article that analysed their training solely using qualitative methodology [50].

### Course developer

Most telehealth training curricula were developed within a university by health-discipline related faculty or staff (*n* = 27, 53%). Many telehealth training curricula were also developed in collaboration between universities and health services, involving a multi-disciplinary group of experts across education and telehealth service delivery (*n* = 15, 29%). One group even collaborated with an educational services platform [9]. Six articles reported their content was developed within a hospital or health service (12%). One article reported that the course content was developed by the federal Ministry of Health [42], while another was developed by their country's professional organisation for rural doctors [68]. One article did not specify who developed the content; however, authors were from a university and a public health service provider [44].

### Health discipline and use

The largest proportion of articles described telehealth courses that were not specific to a discipline or applicable across multiple specialties (*n* = 18, 35%). Targeted areas in other articles included nursing (*n* = 6, 12%), paediatrics (*n* = 5, 10%), primary care (*n* = 4, 8%), and internal medicine (*n* = 3, 6%). Less commonly, there were two studies each directed specifically for the following health subjects: mental health, obstetrics and gynaecology, oncology, and pharmacology. Lastly, the following specialities were represented by just one single study each: anaesthesiology, chronic conditions (not specified by the authors), dermatology, ophthalmology, osteopathy, and speech pathology. Two studies (4%) focused on telehealth with interpreters rather than a medical speciality [43,66].

### Targeted audience

Participants of the telehealth training course described were mostly university students (*n* = 33, 65%) from various health-related programs (e.g., third- and fourth-year medical students, nursing students, physician assistant students, pharmacology students). Nine studies (18%) were educating residents, and eight studies (16%) focused on training already established healthcare providers (e.g., rural doctors, allied health clinicians, nurses). One study included a combination of medical students and residents in their course implementation and evaluation [34].

### Telehealth modalities

From the included studies, 8 (16%) had specific features to enhance video consultation skills and competencies [21,37,41,44,51,52,61,62], two (4%) focussed on telephone [48,57], and 3 (6%) mentioned both modalities [27,60,68]. Two studies (4%) focused on multiple telehealth modalities, including teleconsultation, tele-expertise, medical telemonitoring, tele-medical assistance and emergency telemedicine [31,67], while the remaining studies (*n* = 36, 71%) mentioned telehealth or telemedicine overall, without specifying the modality.

### Learning objectives and course content

Approximately two-thirds of studies (*n* = 33, 65%) listed explicit learning objectives, while the remaining only described course content without reference to specific learning outcomes (*n* = 18, 35%).

By mapping the learning objectives across the AAMC telehealth domains [13], we identified that: 27 (53%) courses addressed patient safety and appropriate use, 22 (43%) covered data collection and assessment, 21 (41%) included communication, 17 (33%) focused on ethical practices and legal requirements, 19 (37%) covered technology, and 16 (31%) addressed access and equity. Only 4 (8%) studies listed all six AAMC telehealth domains [34,47,53,71]. A summary of learning objectives beyond the AAMC telehealth domains is presented below.

Learning objectives commonly focus on building foundational knowledge of telehealth, including understanding its definitions, modalities, benefits and limitations. In the courses analysed, learners were expected to develop competencies in using telehealth platforms effectively, ensuring patient safety, privacy, confidentiality, and compliance with legal and ethical standards (‘netiquette’ [29]) [22,25,29,30,32,34,41,42,44,47,52–54,56,64,67,72]. Some courses placed a significant emphasis on developing clinical and communication skills tailored to virtual care environments, including physical examinations [25,35,41,43,52,53,58,67], conducting history-taking, data collection and assessment, patient education at distance [21,34,35,41,43,52,53,58,60], while maintaining professionalism and empathy through effective ‘webside manner’ [29]. Some programmes reinforced the need for learners to be able to identify situations where an in-person referral was needed [21]. Communication or ‘on-camera etiquette’ [58] included verbal and non-verbal communication skills [52,53,58], trust and rapport building [31,35,42,52], how to develop confidence to engage with patients [60,66] and how to perform certain physical examinations [22,37,41,52].

Some programs also aimed to foster interprofessional collaboration, encouraging teamwork among diverse healthcare providers in virtual settings [31,41,60,67,69]. Additionally, objectives often included addressing health equity, digital literacy, and the unique needs of vulnerable populations, such as children, the elderly, and individuals with limited access to technology [22,29,31,34,35,43,47,53,56,60,64,66,69,72]. Some studies also included in their learning objectives, billing, reimbursement and payment criteria for telehealth services [28,32,67].

Course content typically integrated both theoretical and experiential learning (*n* = 35, 69%). Theoretical learning components included online lectures, asynchronous modules, readings, handouts, and interactive multimedia materials. Experiential training was delivered through observations of telehealth visits, standardised patient encounters, supervised role-playing or simulations, and virtual or tele-OSCEs, allowing learners to apply theoretical knowledge in realistic scenarios. Fourteen studies (27%) did not describe any experiential learning [22,23,27,33,38,42,45,46,54,55,58,61,64,66] while only 1 (2%) did not describe any theoretical learning [53]. Learners exposed to experiential learning were guided to reflect on their telehealth experiences, evaluate their competencies, and understand the broader implications of telehealth on healthcare delivery and patient outcomes.

Interprofessional education is facilitated through collaborative exercises, and many curricula were embedded within existing academic programs, ranging from short workshops to longitudinal courses, up to 16 weeks [47], or telehealth modules embedded in a longer course (18 months) [30]. Feedback mechanisms such as debriefings [21,51,52,60], peer evaluations [24,30,42], and faculty assessments [24,57,60,62] were strategies adopted to reinforce learning. Assessment strategies included quizzes [34,38,42], reflective assignments, [29,34,41,60,69], submission of Subjective, Objective, Assessment and Plan (SOAP) notes [72], mini clinical evaluation exercise (mini-CEX) [21,62], and Objective Structured Clinical Examination (OSCE) [25,40], which ensured comprehensive skill development.

### Course evaluation

A total of 46 studies (90%) assessed their courses from the participants' perspective, with 44 (86%) indicating positive feedback. The primary outcome reported in 21 studies (41%) was increased confidence across multiple domains essential for conducting telehealth appointments, including effective patient engagement via technology, performing physical examinations remotely, and appropriately documenting telehealth visits. Additional outcomes included enhanced knowledge (*n* = 15, 29%), skills (*n* = 6, 12%), competence (*n* = 5, 10%), satisfaction (*n* = 4, 8%), and comfort (*n* = 4, 8%) in conducting telehealth consultations after taking the training. One study found that course participants mainly disliked the human factors and user-technology issues in telehealth [54]. A study found that courses rated highest gave students more direct patient care and supervised telehealth experiential activities [60]. While five studies did not formally evaluate their courses [31,39,63–65], two nonetheless noted positive participant feedback, specifically citing increased confidence and proficiency in conducting telehealth following course completion [31,65].

## Discussion

This study systematically reviewed original studies related to telehealth courses developed since the COVID-19 pandemic, highlighting significant variability in course design, content, learning objectives, and delivery methods. Most programs aimed to build basic knowledge and skills in telehealth, emphasising patient safety, communication, technology use, and ethics. The studies were primarily from the USA and targeted diverse health disciplines and learner groups, including undergrad and postgraduate students as well as clinicians, with curricula commonly created by universities, sometimes in partnership with healthcare providers. Learning objectives often addressed key areas such as technology and communication, though few covered all telehealth competencies comprehensively. While many courses combined theory and practical experience, some lacked hands-on approaches. Assessment of learning varied, including methods such as quizzes and clinical evaluations.

Our review demonstrates a significant rise in research focused on telehealth education and training since the onset of the COVID-19 pandemic, as compared to earlier periods [16,17]. This trend reflects increasing interest in advancing telehealth-related educational initiatives. Although the pandemic may have been the catalyst for this growth, enhancing the healthcare workforce's digital health competencies can only be advantageous, particularly since health systems continue to adopt telehealth as an effective, safe and feasible modality of healthcare delivery [73].

This growing interest may also be linked to policy changes, particularly in relation to health workforce development. In Australia, examples of policies and professional body guidelines that demonstrate growing interest in telehealth education and training include permanent Medicare telehealth funding, Australia National Digital Health Strategy, and updated AHPRA telehealth guidance [74–77]. Studies had shown that the lack of relevant policy can be a major barrier [78]. Recently, many international medical and allied health education authorities have recognised telehealth as an essential part of health workforce development [13,14,79]. Endorsement, recommendation and accreditation of telehealth as an important element in health curricula may have prompted the rapid growth of telehealth education and training.

More broadly, recent literature on digital health education in Australia highlights both progress and ongoing gaps in the field. A recent review found that only 33% of Australian universities currently offer dedicated digital health courses, while more than half included digital health topics within broader programs such as public health, data science, or health administration [80]. Most of these offerings (98%) are at the postgraduate level [80], contrasting with our findings that mostly targeted undergraduate students. Since 85% of studies in our review are from North America, this comparison suggests that undergraduate students in Australia have limited early exposure to digital health concepts. This poses challenges for the healthcare workforce, since a large proportion of Australian clinicians lack postgraduate qualifications [81]. Finding room for new content in already established health professional curricula is likely challenging but we hope this review raises the question for educators of whether it may be possible to include more telehealth and digital health training earlier in degree programs, considering its widespread use in healthcare.

One notable challenge in advancing telehealth education is the development of discipline-specific curricula that address the unique needs and competencies required across various clinical areas and health specialties. Developing tailored content can be resource-intensive and complex, given the diversity of clinical practices combined with the evolving nature of telehealth. An alternative and potentially more efficient approach would be to establish a generalised telehealth curriculum framework that covers the foundational principles, including safety, ethical, legal, financial, practical (i.e., physical examination), technical and equity requirements alongside core competencies relevant to all health professionals, such as the AAMC [13]. A digital health capability framework [14] or an outcomes-oriented model like the Quintuple Aim of Healthcare—a framework designed to optimise healthcare systems by focusing on five aims: population health, patient experiences, costs, clinician well-being, and health equity—can help identify and organise core competencies for healthcare professionals' education and training [82]. But as telehealth becomes standard practice, it's crucial to clearly define and consolidate the skills healthcare professionals need to leverage beyond the technologies to enhance health care delivery and outcomes. For example, when it comes to addressing Health Equity, the fifth domain of the Quintuple Aim of Healthcare [82], training in telehealth services with interpreters for people with diverse linguistic background is essential [43,66].

With the overarching structure of a telehealth curriculum framework, discipline-specific case studies and practical examples can be embedded to illustrate the distinct applications, challenges, and limitations faced by different specialties [25,83]. This strategy allows for both standardisation and customisation, ensuring that all students or clinicians gain a strong baseline of telehealth knowledge and skills while also developing an understanding of how telehealth practices must be adapted to suit the nuances of their chosen field or patient needs. Such an approach may facilitate broader curriculum adoption and promote interdisciplinary understanding, while still addressing the specific learning needs of diverse healthcare disciplines.

Our findings demonstrated variability in the comprehensiveness and focus of learning objectives with only a small proportion of courses addressing all six AAMC telehealth domains [13]. While pre-pandemic literature on telehealth education and training emphasised primarily technological competencies [16], more recent included studies are showing inclusion of foundational knowledge, patient safety, appropriate use, communication skills, privacy and legal requirements. The need for a wider range of essential competencies in virtual care delivery [84,85] was likely recognised after the rapid and large adoption of telehealth which revealed gaps in the skills and abilities required to provide these services effectively and safely. However, not all curricula provide thorough coverage of these expanded competencies. Recent literature continues to highlight these shortcomings, noting that despite advancements in basic telehealth competency training, a comprehensive approach encompassing all domains for both undergraduate and postgraduate learners remains insufficient [80,81,86].

Our review focused on training in telehealth consultations, however, recent research on the broader area of digital health training has also highlighted the need for a curricula to have an increased focus on emerging areas like artificial intelligence and cybersecurity. Other suggestions for improving training in the field have been using standardised curriculum frameworks and accreditation standards, improving regional distribution of programs, and having stronger pathways from education to workforce integration [80].

The integration of both theoretical and experiential learning approaches is another key finding aligning with contemporary educational practice. Foundational literature on education underscores the importance of combining knowledge gain with hands-on experiences for more effective learning [87,88]. However, nearly a third of included studies did not include any simulation, standardised telehealth consultations, or observation of telehealth encounters. While organising clinical placements can be challenging for tertiary institutions, experiential learning should be encouraged as it is essential for reinforcing theory and developing real-world skills. This underscores the need for more practical and reflective elements in telehealth training with adequate opportunities for students to practise telehealth skills in realistic scenarios.

### Strengths and limitations

The main strength of this systematic review lies in its comprehensive overview of the latest developments in telehealth curricula across all health disciplines and stages of health professional careers from undergraduates up to employees enrolling in CPD courses. The extensive expertise of the authors in both digital health education and research is also a strength, across not only Australia but multiple other countries. This experience strengthens the credibility and depth of the review, as the authors are well-versed in how digital health education is designed, implemented, delivered and researched in different settings and to attend to different needs.

It is possible that some examples of established telehealth curricula implemented prior to COVID-19 pandemic may have also been useful to include, however, with rapid changes in technology and hybrid models of care, we felt this was less relevant. Also, it is important to acknowledge that studies published in 2020 may not fully capture COVID-19-related changes, as many likely reflect practices already in place at the onset of the pandemic. Further, the generalisability of our results may be limited as the included studies were mostly from the USA. This may be in part due to only including articles available in English, however, the domination of the Global North in academic peer-reviewed literature is an established inequity across disciplines [89]. That said, included studies from non-English speaking countries (Brazil, Israel, Japan, Malaysia) accounted for 8% of our sample. As international interest and investment in telehealth continue to grow, future reviews should aim to incorporate a broader range of literature, including non-English publications, to enhance global applicability and address existing disparities in academic representation. This would support more equitable and comprehensive curriculum development, ensuring that telehealth education evolves in line with both technological advancements and the varied needs of health systems worldwide.

We acknowledge that health professional education and training in telehealth is just one side of the equation. Improving consumers digital literacy through their own set of training is urgent to foster trust, adoption and sustainability [90,91], a topic which was outside the scope of this review. We also acknowledge that telehealth, (i.e., video and phone consultation with healthcare professionals) is just one modality of many under the digital health umbrella, which is transforming drastically and quickly how healthcare is delivered. Technologies like AI, machine learning, and blockchain may be important to upskill practitioners in alongside telehealth, aligned with current digital health capabilities frameworks [14].

## Conclusion

This systematic review showed that telehealth education and training has expanded since the COVID-19 pandemic. However current curricula are lacking experiential learning and hands-on training. Future telehealth programs can base course content off expert-consensus-based frameworks, such as the AAMC's, to ensure comprehensive coverage of topics. More telehealth curricula should incorporate observation of live or simulated telehealth consultations, where hands-on encounters are not possible. For efficient use of resources, standardised and widely applicable telehealth training curricula should be promoted for use across disciplines, with speciality or site-specific models and cases incorporated by relevant training providers where required.

## Data Availability

Data sharing is not applicable to this article as no new data were created or analysed in this study.

## Appendix A:

### Search strategy exemplar

**Table:**

Steps	PCC elements	Search strategy
1	**Population:** Clinicians/Clinicians in training	**Title/Abstract terms**: *Allied health* OR *Dentist** OR *Clinician** OR *Dietitian** OR *Doctor** OR *General practitioner* OR *Graduate medical* OR *Healthcare staff* OR *Healthcare worker* OR *Medical personnel** OR *Medical profession** OR *Medical staff** OR *Midwife** OR *Midwiv** OR *Nurse** OR *Nursing** OR *Pharmacist** OR *Pharmacist** OR *Physician* OR *Postgraduate* OR *Radiographer** OR *Resident** OR *Surgeon* OR *Therapist** OR *Undergraduate* **Medical subject headings**: *Health personnel* OR *Internship and residency* OR *Students, health occupations*
2	**Concept:** Telehealth	**Title/Abstract terms**: *Digital health* OR *Digital medicine* OR *E-health* OR *Econsult** OR *Econsultations* OR *Ehealth* OR *Electronic consultation** OR *Mhealth* OR *Mobile app** OR *Mobile health* OR *Mobile health app** OR *Phone consultation** OR *Remote consultation** OR *Remote patient monitoring* OR *Telehealth* OR *Tele-care* OR *Tele-health* OR *Telecare* OR *Teleconsult* OR *Telemedicine* OR *Telemonitoring* OR *Telephone consultation** OR *Video consultation** OR *Virtual care* OR *Virtual medicine* **Medial subject headings**: *Digital health* OR *Remote consultation* OR *Telemedicine*
3	**Context:** Medical education	**Title/Abstract terms:** *Continuous education* OR *Courses* OR *Curricula* OR *Curriculum* OR *Health education* OR *Higher education* OR *Medical education* OR *Professional development* OR *Tertiary education* OR *Training** **Medical subject headings:** *Curriculum* OR *Education, continuing* OR *Education, dental* OR *Education, graduate* OR *Education, medical* OR *Education, nursing* OR *Education, pharmacy* OR *Education, public health professional* OR *Inservice training*
4		**Data collection was March 2020-onwards**
5		**1 AND 2 AND 3 AND 4**
